# Estimating Contact Process Saturation in Sylvatic Transmission of *Trypanosoma cruzi* in the United States

**DOI:** 10.1371/journal.pntd.0000656

**Published:** 2010-04-27

**Authors:** Christopher Kribs-Zaleta

**Affiliations:** Mathematics Department, University of Texas at Arlington, Arlington, Texas, United States of America; New York University School of Medicine, United States of America

## Abstract

Although it has been known for nearly a century that strains of *Trypanosoma cruzi*, the etiological agent for Chagas' disease, are enzootic in the southern U.S., much remains unknown about the dynamics of its transmission in the sylvatic cycles that maintain it, including the relative importance of different transmission routes. Mathematical models can fill in gaps where field and lab data are difficult to collect, but they need as inputs the values of certain key demographic and epidemiological quantities which parametrize the models. In particular, they determine whether saturation occurs in the contact processes that communicate the infection between the two populations. Concentrating on raccoons, opossums, and woodrats as hosts in Texas and the southeastern U.S., and the vectors *Triatoma sanguisuga* and *Triatoma gerstaeckeri*, we use an exhaustive literature review to derive estimates for fundamental parameters, and use simple mathematical models to illustrate a method for estimating infection rates indirectly based on prevalence data. Results are used to draw conclusions about saturation and which population density drives each of the two contact-based infection processes (stercorarian/bloodborne and oral). Analysis suggests that the vector feeding process associated with stercorarian transmission to hosts and bloodborne transmission to vectors is limited by the population density of vectors when dealing with woodrats, but by that of hosts when dealing with raccoons and opossums, while the predation of hosts on vectors which drives oral transmission to hosts is limited by the population density of hosts. Confidence in these conclusions is limited by a severe paucity of data underlying associated parameter estimates, but the approaches developed here can also be applied to the study of other vector-borne infections.

## Author Summary

The parasite *Trypanosoma cruzi*, transmitted by insect vectors, causes Chagas' disease, which affects millions of people throughout the Americas and over 100 other mammalian species. In the United States, infection in humans is believed rare, but prevalence is high in hosts like raccoons and opossums in the southeast and woodrats in Texas and northern Mexico. The principal U.S. vector species appear inefficient, however, so hosts may be primarily infected by congenital transmission and oral transmission caused by eating infected vectors. Mathematical models can evaluate the importance of each transmission route but require as inputs estimates for basic contact rates and demographic information. We estimate basic quantities via an exhaustive review of *T. cruzi* transmission in the southern and southeastern U.S., and use properties of mathematical models to estimate infection rates and the threshold (saturation) population-density ratios that govern whether each infection process depends on host or vector density. Results (based on extremely limited data) suggest that oral transmission is always driven by host density, while transmission to vectors depends upon host density in cycles involving raccoons and opossums, but upon vector density in cycles involving woodrats, which live in higher concentrations.

## Introduction

Since the Brazilian physician Carlos Chagas discovered the parasite *Trypanosoma cruzi* in 1909, much research has been devoted throughout the Americas to the study of its transmission and control, primarily in the domestic and peridomestic settings in which it is passed to humans, via triatomine insect vectors of the subfamily Triatominae (Hemiptera: Reduviidae). Although control measures have succeeded in preventing new infections among humans in some areas of Brazil, Uruguay, Chile, and Argentina, the parasite, which is native to the Americas, remains endemic in sylvatic settings as far north as the United States, being limited only by the habitats of the several vector species. In each region, the epidemiology of sylvatic *T. cruzi* transmission differs in important particulars, as each host and vector species has certain peculiarities—behaviors or immunities—which have led to adaptations in the ways by which the infection is maintained.

In the United States, sylvatic hosts (which rapid urbanization often brings into peridomestic settings) include primarily raccoons (*Procyon lotor*) and opossums (*Didelphis virginiana*) in the southeast and woodrats (*Neotoma micropus*) in Texas, although dogs and armadillos have also been cited as significant, and the parasite is also found in skunks, foxes, squirrels, mice, and other *Neotoma* spp. (Vectors do feed upon birds, reptiles and amphibians as well, but these are refractory to *T. cruzi* infection [1], and hence incompetent hosts.) There are over 130 species of triatomine vectors, of which 11 are known to inhabit the southern United States, 8 of them in Texas [2]. Two of the most important in the southeastern U.S. [2], [3] are *Triatoma sanguisuga*, found from central Texas all the way east to islands off the Atlantic coast, and *Triatoma gerstaeckeri*, associated primarily with woodrat nests and domestic settings from central Texas south into Mexico as far as the state of Queretaro [4]. In addition, there are different strains of *T. cruzi* circulating in these populations. Strains are classified within six major groups known as Type I and Type IIa through IIe. Of these, only Types I and IIa are known to circulate in the United States [5], and it is widely believed (primarily from experiments in mice, e.g., [6]–[8]) that the strains circulating in the U.S. are less virulent than those in Latin America, where the incidence of Chagas' disease in humans is much higher: an estimated 16–18 million people (only a handful of autochthonous cases have been diagnosed in the United States [9], though it has also been estimated that as many as half a million people in the U.S. may harbor the parasite, due to migration from Latin America). Among sylvatic hosts in the United States, raccoons and other placental mammals are associated with Type IIa infections, while opossums are associated with Type I infections [5].

*T. cruzi* may be transmitted in a number of ways. Historically, the primary infection route, especially in South America, has involved the vector's feeding process, in which a bloodmeal from an infected host can transmit the parasite to the vector, where it lives in the insect's gut, and defecation by an infected vector on the host following the bloodmeal can result in stercorarian transmission to the host. In sylvatic hosts this may occur when the animal scratches the bite and inadvertently rubs the parasite-contaminated matter into the lesion. However, among humans there have recently been other transmission avenues of greater concern: the parasite can be passed from one human to another through blood transfusion and organ transplants, congenitally from mother to child through the placenta, and oral transmission by consumption of food contaminated by vectors has been blamed for outbreaks in South America. In fact, these avenues of transmission may also be important for sylvatic hosts as well: vertical (congenital) transmission has been verified experimentally among rats [10] and supported by circumstantial evidence among lemurs [11] and other animals, and oral transmission to hosts through their predation upon vectors (raccoons, opossums, and even woodrats are opportunistic feeders that commonly include insects in their diets) has even been suggested by some [12], [13] to be the primary means of *T. cruzi* transmission to hosts in some cycles in the U.S. Indeed, *T. sanguisuga* and *T. gerstaeckeri* are known to be so cautious in their feeding behavior as to avoid climbing up entirely onto hosts during feeding [3], and often defecate 30 minutes or more after feeding ends, making them likely to be rather inefficient at stercorarian transmission to hosts. Both oral and stercorarian transmission to hosts, however, as well as bloodborne transmission to vectors, may be amplified by changes in vector behavior caused by infection with *T. cruzi*. Many disease vectors are known to increase their feeding rate when infected, due to parasites building up inside their digestive tracts and impeding feeding. This behavior has been verified for one species of triatomine vector and trypanosome [14], but not documented for Chagas vectors and *T. cruzi*.

Many of the still-unanswered questions regarding sylvatic *T. cruzi* transmission cycles may be exceptionally difficult to address through direct observation in the laboratory and field: for instance, which of the several transmission pathways is really dominant in each cycle? (We may think of a cycle as a specified host, vector, parasite strain, and geographic region, although in practice such cycles communicate with each other, primarily via vector dispersal.) Mathematical models have proven a useful tool in many fields, including ecology and epidemiology, as they can describe, predict, and provide evaluation measures for phenomena which may be difficult to observe directly. Population biology models consisting of dynamical systems (usually systems of differential equations, see, e.g., [15]), which describe the spread and growth of populations over time, have made notable contributions to disease control beginning notably with Ronald Ross's study of malaria transmission in the early 1900s [16], for which he later won the Nobel Prize. Such mathematical modeling of *T. cruzi* transmission has to date involved primarily household-based modeling of vector infestations and human infection (but see below for a notable exception), although in the past decade geospatial models have been developed to describe vector distribution, disease risk, and relevant ecological niches [2], [17].

The ability of mathematical models to explain and predict depends not only on the underlying assumptions about the biological processes (demographic, infection-related and other) used to construct them, but also on knowing the values of certain fundamental parameters, most of which can be observed directly: information such as average lifespan, population density, or the probability of a host becoming infected from consuming an infected vector. For instance, the ability of a given population to invade or persist in a habitat often depends on threshold quantities such as a reproductive number (which can be calculated in terms of these fundamental parameters) being above or below a critical value. The best-known of these is the *basic reproduction number* for an infection or population [18], [19], denoted 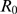, which typically signals persistence of the population precisely when 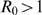. In practice, however, the parameters' values for a given transmission cycle change seasonally, from one region to another, and even from study to study (especially if sample sizes are small). As a result, the critical link between theoretical models and empirical data provided by parameter estimation requires a broad perspective and familiarity with a range of empirical literature.

As noted above, numerous mathematical modeling studies have been published of *T. cruzi* transmission to humans (e.g., [20]–[22]), but almost none have been published on the sylvatic transmission cycles that maintain the parasite. Decades of studies have established details of the life cycles of *T. cruzi* hosts and vectors in the United States, but studies focused on measuring infection parameters are only just beginning to appear (e.g., [13]). Mathematical models can bridge this gap by facilitating calculation of these parameters using enzootic prevalence observations together with known information on the life histories of host and vector species. The aims of the present study are to estimate values for those measures of host and vector life histories and *T. cruzi* infection which have been observed directly in the literature via an extensive review, and then to illustrate a method by which other key infection-related parameters can be calculated using mathematical models.

One of the important aspects of the sylvatic *T. cruzi* transmission cycle which models can help investigate is density dependence in the infection rates. (In this paper the term “rate” refers to a frequency per unit of time at which an event occurs. The term “proportion” will be used to refer to ratios which do not involve time, such as disease prevalence.) Infectious disease transmission is driven by contact processes between susceptible and infective individuals, and sylvatic transmission of *T. cruzi* in particular depends on both the vector-initiated process of taking bloodmeals and the host-initiated process of predation on vectors. The rates at which these two contacts occur depend in part on the host and vector population densities, and in part on the *ratio* of those densities, due to the saturation that occurs when this ratio is too high or too low. That is, the *per capita* contact rate is a function of the vector-host density ratio, so that the total contact rate is the product of this function and the respective (host or vector) density. Ratio-dependent contact rates, which were used in epidemiological models as early as Ross's classic malaria model [16], are also a well-established notion in the study of predator-prey systems [23], [24], and the present study will illustrate how these correspond to the density-dependent effects observed in the transmission of *T. cruzi* (e.g., [25]).

Saturation in contact processes—the notion that given rates can increase only up to a certain point—has also been studied extensively in the contexts of both predator-prey systems (e.g., [26]) and mathematical epidemiology (leading to the distinction between mass-action incidence for low densities and standard incidence for high densities). Predation and infection are superimposed in the transmission of vector-borne infections, and empirical studies [25], [27] have observed a corresponding density dependence in which per-vector biting rates *decrease* at high vector-host ratios. Per capita contact rates thus increase with the density ratio only up to a certain limit, so that the total contact rates (per capita rates multiplied by host or vector density) then become functions of one density or the other alone. When the ratio of vectors to hosts is low, hosts are plentiful relative to vectors, so on the one hand each vector can feed as often as it wants (that is, at its preferred feeding frequency), but on the other hand an average host has a hard time finding vectors to consume, making both contact processes limited by the number of vectors. When the ratio of vectors to hosts is high, however, there are not enough hosts upon which for the vectors to feed at their desired frequency (requiring them to find other blood sources), but the hosts are able to eat until reaching satiation, so that both contact processes are limited by hosts. One recent theoretical study [28] developed a mathematical model for sylvatic transmission of *T. cruzi* and determined that the way in which the two contact processes saturate can affect not only vector population densities but also whether the infection cycle persists. Another study [29] found that such a model coupled to one involving human infection explained observed domestic prevalence data better than a model of exclusively domestic transmission. In order for a mathematical model to predict the rate at which new infections occur, it is necessary to derive quantities such as threshold density ratios from empirical data, so as to understand in what phase of saturation the causative contact processes are operating. This paper presents a way to do so.

This paper derives estimates for the key biological parameters needed to model sylvatic *Trypanosoma cruzi* transmission cycles in Texas and the southeastern United States involving raccoons, Virginia opossums, woodrats, and the two vector species *Triatoma sanguisuga* and *Triatoma gerstaeckeri*. Many of these parameters can be estimated directly via an extensive literature review, but infection and contact rates will be estimated indirectly using estimated prevalence levels and a few properties of some relatively simple dynamical population models. The results will also be used to address the issue of saturation in the two infectious contact processes. The intention is to provide well-informed direct estimates of as many quantities as possible and a method for computing other estimates which can be applied to models designed to address a broad spectrum of questions.

## Methods

An exhaustive literature review was used to derive estimates for basic demographic information on host and vector species, as well as those epidemiological parameters for which direct estimation is possible. The review initiated with a Medline search on “Triatoma sanguisuga”, “Triatoma gerstaeckeri”, or “Trypanosoma cruzi”, together with “United States”—or, for general demographic information on hosts, keywords used were “raccoon”, “opossum” and “woodrat”. From the over 1000 resulting articles, only those (approximately 80) which reported data on one of the quantities estimated in the Results section of this paper were kept. The vast majority of the papers discarded focused exclusively on genetics or microbiology, rather than population biology, and were discarded from the title and abstract; the full text of all other articles was examined for relevant data. Results were found (and kept) in English, Spanish, and Portuguese. References in the sources were then checked manually as well. Gray literature was not specifically sought except for non-Chagas-related demographic information on host species not identified in scientific literature, but was checked when it appeared as a reference in another source. Additional references were added at reviewers' suggestions.

Well-established properties of nonlinear dynamical systems models were then used to estimate infection rates based on prevalence and known parameters, and to frame the estimation of the threshold population-density ratios that determine whether host or vector population densities drive each type of infectious contact. (Specific simple models are used as illustrations in the Results section, but the approach outlined can be applied to a wide variety of dynamical systems, and results are not meant to be limited to the models given.) Models were used (and will be discussed) only where necessary to help estimate relevant quantities.

In every case, epidemiological quantities were estimated as time-averaged values over an entire year, in order not to allow seasonal fluctuations (which impact both host and vector populations significantly) to prevent study of endemic steady states and prevalence.

## Results

### Demography

Basic demographic information on host and vector species is necessary for all modeling of *T. cruzi* transmission cycles. Numerous studies have published data supporting the estimation of average lifespans for raccoons [30]–[34], opossums [12], [34], [35], and woodrats [36, and references therein]; reproductive rates for raccoons [30]–[32], opossums [34], [37], and woodrats [37]; population densities for raccoons [32], [38]–[47], opossums [40], [41], [48], and woodrats [36], [49], [50]; average lifespans for *T. sanguisuga* [3], [51] and *T. gerstaeckeri* [3], [52], [53]; reproductive rates for *T. sanguisuga* [3], [12], [51] and *T. gerstaeckeri* [3], [53]; and, in a single case, vector population density [54]. Discussion and development of estimates for these quantities are provided in Text S1. Table 1 summarizes these estimates (including SI equivalents) for the demographic parameters of each species.

**Table 1:** Estimates for demographic parameters.

Species	Death rate 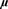	Growth rate 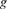	Density carrying capacity 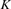	Equilibrium density 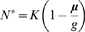
Raccoon	0.40/yr	0.9/yr	0.144 rac/acre (35.6 rac/km 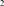 )	0.08 rac/acre (20. rac/km 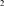 )
Opossum	0.83/yr	4.7/yr	0.0497 opo/acre (12.3 opo/km 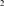 )	0.0409 opo/acre (10.1 opo/km 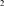 )
Woodrat	1/yr	1.8/yr	21 rats/acre (5200 rats/km 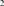 )	9.3 rats/acre (2300 rats/km 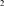 )
*T. sanguisuga*	0.271/yr	33/yr	129 vec/acre (31900 vec/km 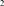 )	128 vec/acre (31600 vec/km 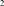 )
*T. gerstaeckeri*	0.562/yr	100/yr	129 vec/acre (31900 vec/km 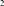 )	128 vec/acre (31600 vec/km 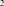 )

### Direct estimation of infection-related parameters

Vertical transmission of *T. cruzi* has been widely documented in humans, and estimated to occur with frequency between 1 and 10 percent in Latin America [55]–[58]. Because the parasite is transmitted through the placenta and blood supply to the fetus, vertical transmission is possible among placental mammals, but it is generally not believed to occur among marsupials. A study in Venezuela found a vertical transmission rate among Wistar rats (*Rattus norvegicus*) of 9.1% for a strain of *T. cruzi* isolated from dogs, but none at all for a strain isolated from humans [10]. Another study in Georgia (USA) found that a Type IIa strain of *T. cruzi* isolate from Georgia was twice as likely to be vertically transferred in mice as a Type I isolate from South America [11]. In the absence of any data on vertical transmission among raccoons, we might reasonably estimate that Type IIa strains are transmitted congenitally roughly 10% of the time (as a proportion, 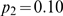), with Type I strains transmitted as much as an order of magnitude less frequently (say 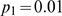).

There is almost no published data on rates of oral infection with *T. cruzi* (which could be estimated directly by multiplying the predation rate of hosts upon vectors by the probability of infection following consumption of an infected vector), although the possibility of oral transmission has long been documented. Olsen et al., writing in the early 1960s, referenced a “postulate” that oral transmission was the primary route of infections for opossums in Alabama, with insects consisting of 43% of opossums' diet by mass, and 60% by volume [12]; Roellig et al. recently extended this notion to include raccoons as well [13]. One recent source wrote, “Animals can easily become infected with *T. cruzi* when an infected triatomine bug is ingested.” [59] However, despite a significant body of research on *what* raccoons, opossums and woodrats eat, a literature review revealed no data on *how much* (or how often) they eat (in order to estimate predation frequency). Rabinovich et al. [60] observed 33 instances of predation when each of 13 female white-eared opossums (*Didelphis albiventris*) was placed with 10 infected *Triatoma infestans* for a day, but the rather high predation rate estimate that would result from this data is skewed by the experimental conditions, e.g., the fact that both opossums and bugs were starved for a period of time prior to the experiment, and the opossums had no other available food. Since predation is opportunistic and there are other insects available to the hosts as well, we will therefore estimate predation to occur for all hosts no more often than one triatomine every 3 or 4 days, which equates to an upper bound of about 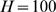 vectors/yr/host. However, it may also be orders of magnitude lower. (Woodrats are of course much smaller than raccoons and opossums, and hence eat less, but vectors are found much more easily in woodrat nests, at least by humans, so we will assume opportunity balances out total volume.)

The probability (or proportion) 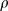 of infection of a host following consumption of an infected vector can be estimated from three experiments in which uninfected hosts were fed vectors infected with *T. cruzi*. Yaeger conducted 11 trials of an experiment in which an uninfected Virginia opossum (*D. virginiana*) was fed two *Rhodnius prolixus* vectors [61] infected with a Type IIe strain; 3 of these trials resulted in infection, yielding an estimate for 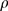 of 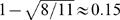. Roellig et al. [13] conducted 2 trials of an experiment in which an uninfected raccoon was fed 3 *R. prolixus* vectors infected with strain IIa; both trials resulted in infection (yielding an estimate for 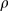 of 1). Finally, the aforementioned study by Rabinovich et al. [60] produced its own estimate of 0.075 for the infection probability of white-eared opossums by eating *T. infestans* infected with an unspecified strain of *T. cruzi* (presumably not IIa); since their experiment combined oral and stercorarian transmission (all 6 of the 13 opossums who ate a bug were also verified to have been bitten by at least one other bug, except for the opossum who ate all 10 of the bugs placed with her), it is impossible to disentangle the raw oral transmission data in a way that can be pooled with the other two experiments. Yaeger's estimate for opossums is precisely twice that of Rabinovich et al., although the difference is not inordinate. Roellig et al.'s data is based on so few trials that no great significance can be ascribed to the resulting high estimate for raccoons, but it is nevertheless suggestive that the probability of oral transmission may vary significantly by host species and by parasite strain (opossums appear not to become infected when exposed to Type IIa *T. cruzi* [62], and hence may be more difficult to infect with any Type II strain)—not to mention vector species—which is entirely consistent with the speculation of some biologists that North American strains may have adapted in response to local conditions. Obtaining a single estimate for opossums requires an assumption that differences due to species (*D. virginiana* vs. *D. albiventris*), vector species, and possibly parasite strain are negligible, in which case we can take a weighted average of 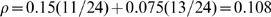. To estimate oral infection probability for raccoons we are left with either the above 100% estimate or else an average across all host species (including opossums) of 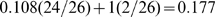.

There is likewise no published research on the extent to which infection with *T. cruzi* increases vector behaviors in *T. sanguisuga* or *T. gerstaeckeri* that promote infection. Añez and East [14] found that triatomine bugs of the genus *Rhodnius*, a common *T. cruzi* vector in South America, probed or bit an average of 6.5 times as often when infected with the parasite *Trypanosoma rangeli* as when uninfected, prior to engorging. This differential behavior may amplify by a factor (say 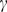) not only the biting rate of infected vectors but also their availability for predation due to increased mobility driven by hunger, so that the effective vector density for infection behaviors is 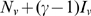 rather than 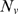. However, D'Alessandro and Mandel [63] found no difference in the feeding behaviors of *R. prolixus* infected by *T. cruzi*. Although such frequencies can be expected to vary widely by species (of parasite as well as vector), it would be consistent with research on South American species to expect no differential behavior in infected *T. sanguisuga* or *T. gerstaeckeri*. In the case where we wish to investigate the possible effects of such an amplification factor, however, it is worth noting Añez and East's value.

Research suggests that in general sylvatic hosts do not suffer mortality from *T. cruzi* infections, even though high mortality rates have been reported for dogs, and the long-term risks have been verified for humans. Also, the mice which die from *T. cruzi* infections in laboratory experiments are often injected with considerably higher concentrations than a single horizontal transmission is likely to produce initially. We may therefore assume (following, e.g., [64]) that in general the sylvatic hosts under study have no significant additional mortality 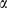 caused by infection with *T. cruzi*.

Table 2 summarizes these parameter estimates. (Table 3 defines additional variables and parameters used in later sections.)

**Table 2:** Directly-estimated, infection-related parameters.

Parameter	Value	Meaning
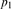	0.01	Vertical transmission proportion for Type I strains
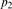	0.10	Vertical transmission proportion for Type IIa strains
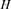	0.1–100 vec./yr/host	(Maximum) per-host vector predation rate
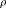	0.177	Proportion of oral infection per infected vector consumed
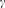	6.5	Behavior amplification factor for infected vectors
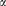	0/yr	Per capita host death rate due to infection

**Table 3:** Model variables and parameters related to infectious contact processes.

Var./Par.	Definition	Units
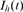	infected host population density (variable)	hosts
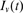	infected vector population density (variable)	vectors
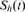	susceptible host population density (variable)	hosts
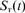	susceptible vector population density (variable)	vectors
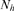	total host population density	hosts
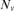	total vector population density	vectors
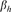	(max.) host infection rate	1/time
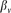	(max.) vector infection rate	1/time
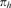	probability of host infection per contact	host/vec/time
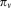	probability of vector infection per contact	vec/host/time
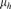 , 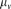	host, vector natural mortality rates	1/time
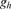 , 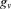	(max.) host, vector reproduction rates	1/time
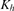 , 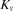	host, vector density carrying capacities	hosts/area, vec/area
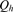	vector-host ratio above which per-host predation saturates	vec/host
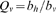	vector-host ratio below which per-vector biting saturates	vec/host
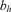	host irritability biting threshold	bites/host/time
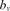	preferred (max.) vector feeding rate	bites/vec/time

### Prevalence

Estimation of the per capita infection rates 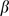 for vector transmission must be made indirectly, as at present there are few published data on both the vector biting rate and the proportion of feedings which result in an infection in each direction (host to vector and vice versa). (Two notable exceptions are [65], which estimated the probability of vector infection per feeding for a specific South American cycle, and [60], which estimated the probability of stercorarian infection of opossums *D. albiventris* at 0.06 [95% CI: 0.023,0.162] per infected *T. infestans* bite). Instead, given the long history of established *T. cruzi* infections in the regions of interest, we shall assume that the parasite has reached endemic equilibrium in the host and vector populations, and use published data to estimate [endemic] prevalence in both host and vector. This will allow us to use the formulas derived from our population dynamics model which express endemic equilibrium prevalence as a function of model parameters, to calculate the infection rates necessary to produce those endemic levels. With prevalence levels and all other parameter values known, it will be possible to solve for the infection rates. But first we must estimate prevalence.

Reported prevalences are given in Tables 4–8 for raccoons, opossums, woodrats, *T. sanguisuga* and *T. gerstaeckeri* in the southeastern United States and northern Mexico. Asterisks (*) denote studies which published paired estimates of host and vector prevalence. For host prevalence, the method of diagnosis is given as [hemo]culture, serology (IFAT = Indirect Fluorescent Antibody Test, IHA = indirect hemagglutination assay), either (both culture and serological tests were performed, and a single positive is reported as positive), blood smear (BS), or xeno [diagnosis]. The dagger 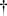 after the citations to Lathrop and Ominsky [66] marks joint prevalence reported for a mixed population of 6 *T. sanguisuga* and 9 *T. gerstaeckeri*.

**Table 4:** Reported prevalences of infection with *T. cruzi* in raccoons (*Procyon lotor*) in the southeastern United States.

Location	Prevalence	Data year(s)	Source	Method
Alabama	5/35 (14.3%)	1961–1963	Olsen et al., 1964, 1966* [12], [87]	culture
Florida/Georgia	9/608 (1.5%)	circa 1958	McKeever et al., 1958 [70]	culture
Florida	2/184 (1%)	1972–1974	Telford and Forrester, 1991 [88]	BS
Florida	4/33 (12%)	1976–1977	Schaffer et al., 1978 [89]	culture
Florida	38/70 (54%)	circa 2009	Brown et al., 2009 [68]	either
Georgia	5/10 (50%)	1977	Schaffer et al., 1978 [89]	culture
Georgia	13/30 (43%)	1994	Pietrzak and Pung, 1998 [90]	culture
Georgia (SE)	50/83 (60%)	1992–1994	Yabsley et al., 2001 [67]	either
Georgia (SE)	12/54 (22.2%)	1992–1994	Pung et al., 1995* [71]	culture
Georgia	51/87 (59%)	1997–2000	Yabsley and Noblet, 2002 [91]	IFAT
Georgia	167/510 (33%)	circa 2009	Brown et al., 2009 [68]	either
Kentucky	25/44 (57%)	2007	Groce, 2008 [34]	either
Maryland	5/400 (1%)	1955	Walton et al., 1958 [92]	culture
Maryland	10/472 (2.1%)	1954–1960	Herman and Bruce, 1962 [93]	culture/BS
Missouri	74/108 (68%)	circa 2009	Brown et al., 2009 [68]	either
North Carolina	3/20 (15%)	circa 1992	Karsten et al., 1992 [94]	culture
Oklahoma	5/8 (62.5%)	circa 1986	John and Hoppe, 1986 [95]	culture
South Carolina	53/134 (40%)	1997–2000	Yabsley and Noblet, 2002 [91]	IFAT
Tennessee (E)	0/6 (0%)	1978	Schaffer et al., 1978 [89]	culture
Tennessee (ctr)	2/3 (67%)	1998	Herwaldt et al., 2000 [96]	culture
Texas (central)	6/25 (24%)	1977–1978	Schaffer et al., 1978 [89]	culture
Texas (south)	0/9 (0%)	1977–1978	Burkholder et al., 1980* [54]	culture/BS
Virginia	0/10 (0%)	1978	Schaffer et al., 1978 [89]	culture
Virginia (north)	154/464 (33%)	2000–2002	Hancock et al., 2005 [97]	IFAT
Virginia	0/12 (0%)	circa 2009	Brown et al., 2009 [68]	either
West Virginia	0/10 (0%)	May 1977	Schaffer et al., 1978 [89]	culture

**Table 5 pntd-0000656-t005:** Reported prevalences of infection with *T. cruzi* in opossums (*Didelphis virginiana*) in the southeastern United States.

Location	Prevalence	Data year(s)	Source	Method
Alabama	17/126 (13.5%)	1961–1963	Olsen et al., 1964, 1966* [12], [87]	culture
Florida/Georgia	93/552 (17%)	circa 1958	McKeever et al., 1958 [70]	culture
Florida	14/27 (52%)	circa 2009	Brown et al., 2009 [68]	either
Georgia (SE)	6/39 (15.4%)	1992–1994	Pung et al., 1995* [71]	culture
Georgia	118/421 (28%)	circa 2009	Brown et al., 2009 [68]	either
Kentucky	21/48 (44%)	2007	Groce, 2008 [34]	either
Louisiana	18/48 (37.5%)	1985–1987	Barr et al., 1991 [98]	culture
North Carolina	1/12 (8.3%)	circa 1992	Karsten et al., 1992 [94]	culture
Texas (central)	8/8 (100%)	1937–1941	Packchanian, 1942 [99]	culture
Texas (south)	63–64/391 (16%)	1957–1958	Eads, 1958 [69]	culture
Virginia	1/6 (16.7%)	circa 2009	Brown et al., 2009 [68]	either

**Table 6 pntd-0000656-t006:** Reported prevalences of infection with *T. cruzi* in woodrats (*Neotoma micropus*).

Location	Prevalence	Data year(s)	Source	Method
Texas (central)	32/100 (32.0%)	1937–1941	Packchanian, 1942 [99]	culture
Texas	161/461 (34.9%)	1950–1951	Eads and Hightower, 1952 [100]	BS
Texas	12/56 (21.4%)	1965–1967	Pippin, 1970* [3]	BS
Texas (south)	7/30 (23.3%)	1977–1978	Burkholder et al., 1980* [54]	culture/BS
Texas (west)	6/13 (46.1%)	1981–1983	Ikenga & Richerson, 1984* [101]	IHA
Texas (west)	7/18 (38.9%)	1981–1983	Ikenga & Richerson, 1984 [101]	IHA
Nuevo León	2/25 (8%)	1990	Galavíz-Silva and Arredondo-Cantú, 1992 [102]	xeno

**Table 7 pntd-0000656-t007:** Reported prevalences of infection with *T. cruzi* in *Triatoma sanguisuga*.

Location	Prevalence	Data year(s)	Source
Alabama	11/181 (6%)	circa 1963	Hays, 1963 [81]
Alabama	6.70%	1961–1963	Olsen et al., 1966 [12]
Georgia (SE)	3/5 (60%)	1992–1994	Pung et al., 1995* [71]
Louisiana	10/18 (55.6%)	2006	Dorn et al., 2007 [9]
Texas	0/10 (0%)	ca 1933–1941	Wood, 1941 [80]
Texas	19.23%	1941–1942	deShazo, 1943 [72]
Texas	4/9 (44.4%)	1942	Davis et al., 1943 [103]
Texas	23/90 (25.5%)	1941–1947	Sullivan et al., 1949 [73]
Texas (south)	50/226 (22%)	1960–1962	Eads et al., 1963 [74]
Texas	6/15 (40%)	1964	Lathrop and Ominsky, 1965 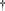 [66]
Texas	33/132 (25%)	1965–1967	Pippin, 1970* [3]
Texas	3/7 (42.9%)	1966	Pippin et al., 1968 [104]
Texas (south)	6/35 (17.1%)	1977–1978	Burkholder et al., 1980* [54]
Texas	10/29 (34.5%)	2005–2006	Kjos et al., 2009 [2]

**Table 8:** Reported prevalences of infection with *T. cruzi* in *Triatoma gerstaeckeri*.

Location	Prevalence	Data year(s)	Source
Nuevo León	26.5%	circa 1990	Galavíz et al., 1990 [105]
Nuevo León	21/75 (28%)	circa 1992	Martínez-Ibarra et al., 1992 [106]
Nuevo León	31/52 (59.6%)	2005	Molina-Garza et al., 2007 [107]
Queretaro	2/9 (22%)	2003–2005	Villagrán et al., 2008 [4]
Texas	3/54 (5.55%)	ca 1933–1941	Wood, 1941 [80]
Texas	92/100 (92%)	1937–1938	Packchanian, 1939 [108]
Texas	30.91%	1941–1942	deShazo, 1943 [72]
Texas	135/450 (29.9%)	1941–1947	Sullivan et al., 1949 [73]
Texas (south)	84/133 (63%)	1960–1962	Eads et al., 1963 [74]
Texas	6/15 (40%)	1964	Lathrop and Ominsky, 1965 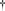 [66]
Texas	46/97 (47.4%)	1965–1967	Pippin, 1970* [3]
Texas (south)	13/49 (26.5%)	1977–1978	Burkholder et al., 1980* [54]
Texas (west)	37/62 (59.7%)	1981	Ikenga and Richerson, 1984* [101]
Texas (south)	24/31 (77.4%)	circa 2003	Beard et al., 2003 [17]
Texas	86/156 (55.1%)	2005–2006	Kjos et al., 2009 [2]

As evidenced by Table 4, dozens of studies have reported prevalence figures for the infection of raccoons with *T. cruzi* in the past fifty years, in states throughout the southeastern quarter of the United States. As observed by several researchers, notably Yabsley et al. [67], the method used to determine infection can have a significant effect on the results: in particular, the parasite is often only found in the blood (by hemoculture or blood smears) during the initial (acute) period of infection, while the immune system takes some time to develop antibodies to *T. cruzi*, so that serological tests like IFAT and ELISA are more likely to detect chronic infections. It is therefore best to use both methods in order to capture both acute and chronic infections. Most studies reported prevalence based only on blood cultures until about ten years ago, and as can be seen in Table 4 there is a marked difference in the prevalences reported based on hemoculture studies as compared to serological or both. Ten of the sixteen blood-based studies reported prevalences of 15% or less (seven of these reported prevalences of 1.5% or less, and the mean of all 16 values is under 20%), whereas apart from a single, small-sample (n = 12) zero value, the studies which included serological results reported a mean of over 50% prevalence.

There is also some notable geographic variation. Infection rates near the central part of the country appear to be relatively high, with studies from Kentucky, Missouri, Oklahoma and central Tennessee all reporting prevalences of well over 50%, with a total prevalence of 106/163 or 65%. On the other hand, the region directly east of that, from the mountains to the Atlantic, has little or no infection: studies from Maryland, Virginia, West Virginia and even eastern Tennessee adjacent to Virginia all report effectively zero prevalence, the exception being a study of raccoons in the suburban area of Fairfax County, Virginia, near Washington, D.C., where increased opportunity for foraging results in a higher raccoon population density.

Prevalence among raccoons in Georgia and neighboring South Carolina ranges from 33% to 60% except for one hemoculture-based study which reported 22%. Pooling these 7 studies yields an overall prevalence of 351/908 or 38.7%, heavily weighted by the large study of Brown et al. [68]. Moving west along the Gulf Coast, there is no data apart from Olsen et al.'s study from eastern-central Alabama in the early 1960s until we reach Texas, where there are only two small studies from 1977–1978. We shall take the figure of 24% from central Texas, rather than that of 0% from south Texas, as being representative of prevalence among raccoons in the central and eastern part of the state.

Examining the reported prevalences for opossums, there is a clear tendency for the studies which used both blood culture and serology to report higher prevalences (see Table 5), with the exception of the early datum from Texas, which was of such a small sample size (n = 8) that it cannot be claimed to be representative. There is nearly an order of magnitude difference in sample size between the three largest studies [68]–[70] and the next largest, and these three show, on the one hand, nearly identical hemoculture-based prevalences between Texas (16%) and Florida and Georgia (17%, consistent with the more recent Georgia figure of 15.4% [71]), and, on the other hand, a prevalence that nearly doubles when both hemoculture and serology are taken into account (28% in Georgia [68]). Although some of the smaller studies suggest that in places the prevalence of *T. cruzi* in opossums may be much higher than this, we shall use Brown et al.'s 28% figure as representative of prevalence in both the southeast and Texas.

The four earliest reported prevalences of *T. cruzi* infection in Texas woodrats are relatively close to each other (ranging from 21.4% to 34.9%, see Table 6) but used hemocultures or blood smears rather than serology, which may imply an underestimate; the two reports from west Texas, both serological, are higher but come from much smaller samples. We shall nevertheless pool the data to obtain an overall prevalence of 225/678 or 33.2%.

Very few studies have reported infection prevalence for the vector *T. sanguisuga* east of Texas (see Table 7). The studies published by Hays, Olsen and their collaborators in the 1960s give prevalences of around 6% in eastern central Alabama, but the two more recent studies in Georgia and Louisiana agree on values an order of magnitude higher. It is likely that infection prevalence does vary by location, but for an overall average we shall pool the two more recent reports, for a total prevalence of 56.5% in the southeast. In Texas, reported prevalences appear to fluctuate within a range of 17% to 44%. Pooling all but the first two studies (since the second gave no absolute numbers) yields an overall prevalence of 135/543 or 24.9%.

Early studies had *T. cruzi* prevalence in the vector *T. gerstaeckeri* varying widely from 5% to 92% (see Table 8), and despite some slight convergence, results continue to fluctuate from 26.5% to 77.4%, even among relatively large (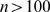) samples (we exclude from further discussion the small sample from Queretaro in central Mexico). Since these studies typically collected vectors from woodrat nests, it is likely that there may be considerable variation in infection proportions from one nest to another. The three reports from the state of Nuevo León, Mexico, just south of Texas, also fit within this range. We will therefore pool all studies for which raw data is given (noting that the rate given in Galavíz et al. is close to that in the study by Martínez-Ibarra et al., on which Galavíz was second author, and that the data in deShazo is likely incorporated into the study by Sullivan et al. given the dates, and the fact that deShazo and Sullivan were the same person), to derive an overall prevalence of 572/1259 or 45.4%.

Note that all collections of vectors in Texas were made from either woodrat nests or peridomestic environments, while collections in the southeast mention association with both raccoons and opossums. This complicates the matter of disentangling the various transmission cycles (for instance, are vectors in raccoon dens in Texas infected at the same level as vectors in nearby woodrat nests?), which may be especially important where different strains of *T. cruzi* are involved, as with opossums (typically infected with type I) and raccoons (typically infected with Type IIa) in the southeast. In the absence of more complete data, however, we can do no better at present than use these figures as applying across hosts in a given habitat.

As a brief aside, we also note reports of prevalence in Texas among the vector *Triatoma neotomae*, uniquely identified with woodrat nests, of 87.5% by deShazo [72], 11/17 (64.7%) by Sullivan et al. [73], 27/31 (87%) by Eads et al. [74], and 2/3 (66.7%) by Burkholder et al. [54], the latter three of which combine to give an overall prevalence of 40/51 or 78.4%, significantly higher than that of most other vector species. As the vector's habitat is confined to one or two regions of Texas, however, we will not consider it further.

Table 9 summarizes these prevalence estimates for Texas and the southeast.

**Table 9:** Estimated average prevalences of principal *T. cruzi* hosts and vectors in Texas and the southeastern U.S.

Species	Texas	Southeast
Raccoon	0.240	0.387
Opossum	0.280	0.280
Woodrat	0.332	N/A
*T. sanguisuga*	0.249	0.565
*T. gerstaeckeri*	0.454	N/A

### Infection rates

Most quantities dealing with the *T. cruzi* infection process itself must be estimated indirectly by inference, since (as illustrated in the previous subsection) little or no published data exists on quantities such as probabilities of infection and even species-specific contact rates. Instead, one can use population models of transmission dynamics to back-calculate the effective infection rates given observed endemic prevalences and the known demographic parameter estimates. The specific calculations and expressions involved are model-dependent—for example, one model may distinguish between oral and stercorarian infection rates for hosts, while another uses a single term with a net host infection rate—but the general idea remains the same: to use equations for the observed endemic equilibrium to solve for the desired parameters. (Note this method assumes that observed infection prevalence represents a steady endemic state.) Table 3 summarizes all model variables and parameters used in modeling discussions in this and the following sections, except for those already defined in Table 2.

To illustrate this technique with a minimum of model parameters, we here consider a scenario with a single host and single vector species, each at a constant population density, and only a single (net) route to infection. The simplest vector infection model has the form
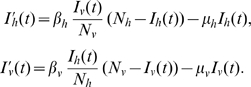
Here 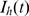 and 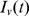 are the densities of infected hosts and vectors, respectively, as functions of time, 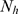 and 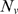 are the host and vector densities as before (here assumed constant over time), 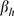 and 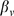 are the respective infection rates, and 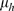 and 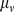 are the mortality rates. In each differential equation the first term describes the rate of new infections, and the second describes removal by natural mortality (we assume no recovery from infection). Here for simplicity we use so-called standard incidence to describe the total infection rates, and defer discussion of saturation in the relevant contact processes until the next section. This model is mathematically equivalent to the classical Ross model for malaria transmission [16], although removal of infected hosts here is due to natural death (not recovery as in Ross's model) and for simplicity the [here constant] vector-host ratio 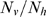 that is explicit in Ross's model has been absorbed into 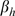 (the following subsection on saturation in contact processes will address how the infection rates depend on this ratio).

If we define proportional infection levels 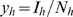, 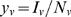, then the equilibrium conditions for this model (setting the time derivatives 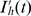 and 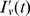 to zero for the steady state) can be written as
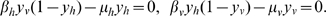
We can solve these equations for the infection rates 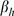 and 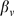, so that in case we know the prevalence levels 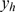, 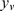 (assumed positive) and also the mortality rates 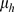, 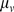, we can calculate the corresponding infection rates:
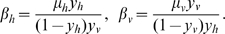
We can apply this result to the transmission cycle between raccoons and *T. sanguisuga* in the southeastern U.S. using the prevalence estimates 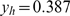, 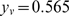 derived in the previous section and the mortality rates 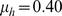/yr, 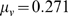/yr from Table 1 (assuming opportunistic host predation on vectors does not significantly impact vector mortality), to obtain
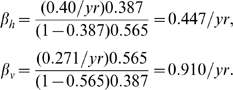
If we instead consider opossums (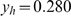, 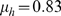/yr) and *T. sanguisuga* in the southeastern U.S., we get instead
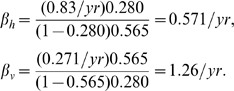
The fact that in both cases 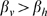 reflects the higher prevalence found in vectors compared to hosts, 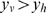, consistent with the observation (e.g., [3]) that *T. sanguisuga* and *T. gerstaeckeri* are so cautious as to rarely walk entirely onto a host, therefore making (stercorarian) transmission to hosts much less likely than transmission to vectors through bloodmeals.

Note that this model assumes no vertical transmission, and treats all transmission routes (here, stercorarian and oral for the host) as one to produce an estimated overall infection rate. Any such distinctions must be made in the model used to derive the infection rates. For instance, if we wish to take into account vertical transmission of *T. cruzi* among placental hosts such as raccoons, then we add a corresponding term 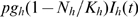 to the equation for 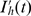 (if hosts are assumed to reproduce according to a logistic law, at a total rate 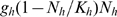):
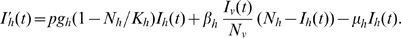
If we assume the host population to have reached its equilibrium value 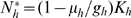, then the new term simplifies to 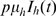, and the differential equation simplifies to its previous form, with 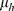 replaced by 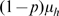:
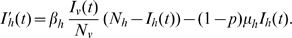
This means that the only change made in the two expressions for infection rates is to multiply 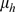 (and hence 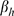) by 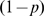:
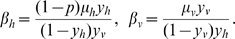
The vector infection rate 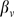 is unaffected, but in the case of raccoons infected with Type IIa *T. cruzi* in the southeastern U.S., the vertical transmission estimate of 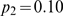 for Type IIa yields an estimated horizontal transmission rate of 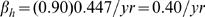.

Similar adaptations can be made for models which distinguish between stercorarian and oral transmission to hosts, or address differential behavior of infected vectors, etc., although sufficiently complicated models may require solving equilibrium conditions numerically once other parameter values are substituted, if closed-form expressions for endemic equilibria are not available.

### Infectious contact processes and saturation

Finally, in order to complete a model description of *T. cruzi* transmission dynamics, it is necessary to address the specific forms of the host-vector contact processes that drive infection: host predation upon vectors, which can produce oral transmission, and vector feeding upon hosts, which can produce bloodborne and stercorarian transmission. Here, too, mathematical models can help identify and articulate the key parameters that determine those forms. Since both types of contact processes are predation-driven, we begin with a brief review of considerations from the well-developed area of predator-prey modeling.

#### Host predation on vectors

Several ecologists and mathematical biologists (e.g., [23]) have argued that the rate of contacts (successful predation) between predators and their prey is most properly a function of the ratio of prey to predators (or vice versa), and this is reasonably the case with predation upon *T. cruzi* vectors, which tend to remain localized close to their food sources (i.e., in the dens or nests of hosts) except for dispersal upon reaching maturity. It is also well-established in the study of predator-prey systems that this contact rate does not increase linearly without bound as the prey-predator ratio increases, but rather it saturates for high values of this ratio, as for low values the predation is limited by the predator's ability to find (and catch) the prey, whereas for high values it is limited by the predator's satiation (desired predation rate) [24], [26]. The per-host predation rate should therefore increase as a function of the vector-host ratio until the ratio reaches a critical level, which we may denote 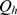 (for host-initiated contact quotient), above which the predation rate saturates, as vectors are then so plentiful that hosts find them readily.

Previous studies of saturation in contact processes including predation [75], [76] have identified so-called Holling Type I saturation, arguably the simplest mathematically, as capturing the greatest variety of dynamics, so we shall assume it here. Under this assumption, the per-predator contact rate has the form 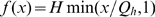 (where the prey-predator ratio 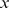 in this case is the vector-host ratio), so that when 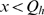 (few vectors per host) 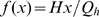 and the rate rises linearly with the vector-host ratio, while for 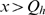 (many vectors per host) the rate is completely saturated at the host's maximum desired predation rate, 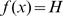. When we substitute the ratio of vector to host densities, 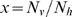, into this form and then multiply by the number of hosts 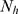 in order to get the total predation rate, we obtain 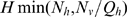. (Note that the prey-predator or vector-host ratio no longer appears explicitly in the expression, because when we multiply the per-predator rate by the predator population 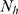 it cancels out the 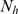 in the denominator of the ratio.) In some sense, 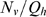 is the maximum number of hosts that can effectively forage for vectors at one time, given the current vector population density. This makes 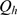 an important parameter to estimate, in order to know which of the two population densities is driving the predation contacts.

Although studies have not been undertaken to estimate the threshold vector-host ratio 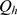, a brief anecdote may help derive the correct order of magnitude. A study conducted in Venezuela in 1976 [77] examined 16 houses with palm-thatched roofs and palm or mud walls for the presence of the vector *R. prolixus*. Researchers spent 4 man-hours searching each house for vectors. Each house was then carefully disassembled the next day bit by bit and any remaining vectors collected. The study found that only 7.1% of the vectors in the houses were found during the initial inspections, with “catchability” increasing with vector size (hardly any early-stage instars were found during inspections, compared to 12.8% of adults). Similar results have been found in other places (e.g., 10–20%, F. Espinoza-Gómez, personal communication). This episode serves to illustrate triatomines' ability to hide in dark, narrow cracks. As a result, if we wish to estimate the vector-to-host ratio sufficient to allow a host to find a vector easily at hand when it is hungry, we may suppose that the vector density should be at least an order of magnitude higher than host density (again assuming only one vector in ten is found easily, despite the differences in the habitations and foraging abilities of sylvatic hosts and humans). We therefore make a very rough estimate of 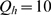 vectors/host, noting that the estimate need not be especially accurate in this case, as the population densities estimated earlier in this paper give a present vector-host ratio of approximately 1600 for raccoons, 3200 for opossums, and 14 for woodrats. In Texas, where vectors in sylvatic settings are found primarily in woodrat nests, this ratio can be applied directly to the host and vector densities, while in the southeast the vectors are distributed among many hosts, so the actual vector-host ratio is somewhat lower. Even so, the actual ratio of vectors associated with raccoons and opossums to the hosts themselves is likely high enough to make them readily available.

#### Vector feeding on hosts

Although the vector feeding process is not strictly speaking predation, it involves a similar type of contact process initiated by vectors, and so one may model it similarly: namely, with a per-predator (here, per-vector) contact rate that is a function of the population density ratio and exhibits Holling Type I saturation as hosts become plentiful. That is, the per-vector biting rate can be described by a function 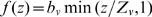, where 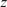 is the prey-predator ratio—here, the host-vector ratio 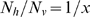
—and 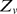 is the threshold density ratio at which saturation occurs, above which the average vector can feed at its preferred rate 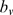 (given in contacts per vector per time), but below which the relative scarcity of hosts constrains the rate at which the average vector can feed on the given type of host (it must then seek other feeding sources). In particular, we assume that an average host can receive bites at a maximum rate 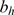, beyond which it successfully defends itself against vectors (including possibly leaving the scene altogether). Then the threshold density ratio is 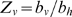.

This idea of a density-dependent feeding rate is supported by recent studies [25], [27]: for instance, it was found that increased *Triatoma infestans* vector density “significantly reduced feedings” on the dogs made available to the vectors, and also tended to reduce the mean bloodmeal size [25]. The authors cited several other studies which support this idea, writing, “In laboratory settings several triatomine bug species frequently showed negative density-dependent engorgement rates on non-anesthetized, unrestrained, small hosts including mice, hamsters, guinea pigs, small chickens and pigeons.” Saturation in the contact rate describes this density dependence in terms of a limitation on the host-vector ratio's ability to increase the per-vector feeding rate.

In order to minimize the number of new variables, we can write the per-vector feeding rate in terms of the (previously-defined) vector-host ratio 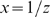, namely 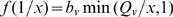, where 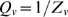. Then the total biting rate produced by all vectors combined is 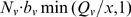; since 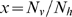 and 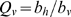, with some algebra this expression can be rewritten in various forms: 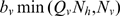, 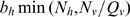, or indeed 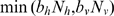. From the first of these three, one can see that 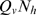 is thus the maximum vector density at which the vectors can still feed on hosts at the desired frequency, and beyond which they must turn to other sources (such as incompetent hosts like birds) for bloodmeals or, in the case of nymphs, parasitize adults of their own species by feeding on the body juices of engorged adults (between the distended sclerites, without apparent harm to the adults, see, e.g., Elkins [78]). From the second form, one can identify 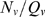 as the minimum host density needed in order for vectors to feed at the desired frequency. The last form, 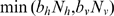, can be interpreted as follows. When hosts are scarce, the total vector-feeding contact rate should be proportional to (limited by) the number of hosts but not the number of vectors, i.e., 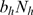 total bloodmeals per unit time (per acre or 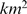). When, on the other hand, hosts are plentiful, vectors can feed at their preferred rate, so the total vector-feeding contact rate should be proportional to vector density and not host density, i.e., 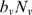 total bloodmeals per unit time (per acre or 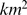).

To determine the rates of new host and vector infections from the rate of vector bloodmeal contacts, we must take into account the probability of infection resulting from a bloodmeal contact where one party (host or vector) is infected with *T. cruzi* and the other is not. We therefore define 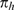 as the probability that such a contact between an infected vector and an uninfected host results in infecting the host, or in deterministic terms the proportion of such contacts that result in an infected host. We likewise define 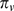 as the proportion of bloodmeal contacts between infected hosts and uninfected vectors which result in an infected vector. Now, in the case where the vector-host ratio is low enough (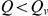, as estimated to be true for woodrats), so that vectors feed at their desired rate, we can calculate the rate of new vector infections as
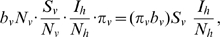
that is, the rate of bloodmeal contacts (in bites/time) multiplied by the proportion of contacts that involve uninfected vectors and the proportion of contacts that involve infected hosts, multiplied finally by the proportion of such contacts that result in an infected vector (in infected vectors/bite). We rename the constant 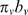 as 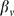, the infection rate estimated indirectly in the “Prevalence” section (in units of 1/time), and indeed the vector infection rate in that section is precisely the one given above.

We can likewise (under this same assumption that 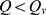) write the rate of new host infections as
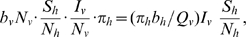
using the fact that 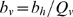. In accordance with the units, we define 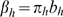 as the baseline host infection rate (1/time), making the total host infection rate 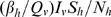. This differs from the simple vector infection model in the “Prevalence” section because the infection rate of hosts is proportional to vector density rather than host density.

However, under the alternate assumption that vectors are plentiful (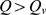, estimated above to be true for larger hosts), the rate of new host infections becomes instead
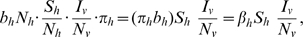
proportional to host density, so that hosts are bitten by vectors at the maximum rate they can tolerate, and any vectors that cannot feed enough on the given hosts are obliged to go elsewhere to feed on other hosts (including at times birds, toads and lizards if necessary). In this case the total rate of new vector infections is given by
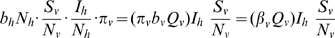
since 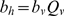.

In this way, regardless of the actual vector-host density ratio 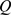, the infection rates need not use 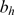 and 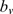 directly, just their ratio 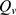 and the effective infection rates 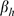 and 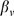 which can be estimated indirectly from prevalence data. We now consider the estimation of 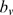 and 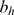 in order to figure 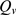.

Published studies on vector feeding behaviors rarely address the preferred feeding frequency 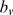 directly. Some authors [3], [79], [80] measured how long vectors could live following a single feeding, but these starvation longevities (e.g., means of 135 days for *T. sanguisuga* and 143 days for *T. gerstaeckeri* in [3]) can serve only to provide lower bounds for 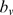. A few studies instead provided vectors regular opportunities to feed (usually at least once per week) and observed what proportion fed on average. In this way Hays [51] found that 73% of field-reared female *T. sanguisuga*, 58% of field-reared males, and 60% of laboratory-reared adults fed twice a week on rabbits in the laboratory; taking an average of 65.5% for field-reared adults yields a frequency of 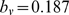 bites/vec/day. This figure is close to the averages that can be calculated from other data given by Hays for adult *T. sanguisuga* [51], [81]: females grown from field-reared nymphs lived an average of 456.5 days, during which time they took an average of 88 bloodmeals, at an overall rate of 0.193 bites/vec/day, while males grown from field-reared nymphs took an average of 80 bloodmeals over 526 days, for a rate of 0.152 bites/vec/day. These figures are considerably lower than the figures obtained from the fieldwork of a group of researchers studying *Triatoma infestans* in Argentina, which gave 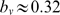 bites/vec/day in one study [82] and monthly averages ranging from 0.30 bites/vec/day to 0.60 bites/vec/day in another [83], but they are consistent with estimates based on the work of another team in Chile [84], of 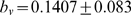 bites/vec/day for *T. infestans* (mean
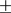
SD) and 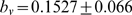 bites/vec/day for *Mepraia spinolai*.

The feeding rates for nymphs, however, are likely much lower, as illustrated by data in Martínez-Ibarra et al. [53] which found that *T. gerstaeckeri* nymphs in Mexico needed an average of 13.2 bloodmeals to mature from egg to adult, but took a mean of 278.6 days to do so (this development time is longer than that given in Pippin [3] but Martínez-Ibarra et al. fed their bugs on rabbits rather than woodrats, to which *T. gerstaeckeri* are specialized); this yields an average feeding rate of 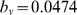 bites/vec/day for *T. gerstaeckeri* nymphs. (In comparison, Pippin found that 5 *T. sanguisuga* nymphs needed an average of 5.4 bloodmeals to molt from first to second instar alone. Martínez-Ibarra et al. found that *Triatoma lecticularia* nymphs needed an average of 14.9 bloodmeals to mature, and *Triatoma protracta* needed 12.6.)

Research on *T. infestans* in Argentina also showed a high degree of correlation between vector biting frequency and temperature, with nymphs feeding at a rate of 0.014 bites/vec/day in July (winter) but 0.442 bites/vec/day in December (summer), and adults feeding at rates of 0.021 bites/vec/day in July (and 0 in May) and 0.610 bites/vec/day in December [85]. For nymphs and adults together a linear regression on temperature in this study gave the prediction 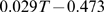 bites/vec/day for *T. infestans*, where the variable 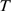 is temperature in degrees centigrade. This same study observed a seasonal shift in the effects of density dependence, as discussed above in terms of Holling Type I saturation: during the warmer months, when vector density was higher, the proportion of recently fed bugs “declined markedly,” while at lower vector densities the vectors apparently fed at their desired rate (for the given temperature).

A detailed description of vector feeding rates, therefore, would distinguish between nymph and adult as well as incorporate variations in temperature and vector density, building models such as the linear regressions in [85]. The most basic possible estimate (a single rate for each species) would have to average over age structure and seasonality. One could calculate a weighted species estimate over each vector lifetime by multiplying the seasonal average biting rate for nymphs by the average proportion of a lifetime a vector spends as a nymph, multiplying the seasonal average biting rate for adults by the proportion of lifetime a vector spends as an adult, and adding the two. Of the two vector species studied in this paper, however, the present review of literature provides only Hays's estimates above for adult *T. sanguisuga* biting rates, and Martínez-Ibarra et al.'s estimate for *T. gerstaeckeri* nymph biting rates. If we make the (perhaps gross) simplifying assumption that the two species' feeding rates are similar, then we might extrapolate (using longevity estimates from the section on vector mortality and reproduction) to estimate the following rate for *T. sanguisuga*:
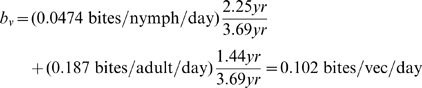
and the following rate for *T. gerstaeckeri*:
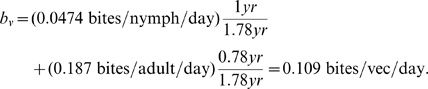
The maximum bite rate 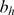 a host can (or is willing to) sustain is even more difficult to estimate, as it has not been studied directly. One study of domestic *T. infestans* infestation in Argentina [82] estimated that humans in one house received as many as 5.52 bites/human/night, although in most houses the average was less than 1. A related study found that chickens (an incompetent host for *T. cruzi* but a common bloodmeal source for vectors) in nearby chicken houses infested with *T. infestans* received an average number of bites per night that varied seasonally from about 1 (April and July) to over 30 (in December) [85]. In general we may consider the host irritability threshold of 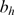 for all three host species (raccoon, opossum and woodrat) to be bounded very loosely between 2 and 40 bites per night (direct observation would no doubt quickly narrow this interval). The upper bound may be even lower if vectors are spatially distributed so heterogeneously that some hosts never encounter vectors (in which case 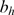 would be reduced by the proportion of hosts that do encounter vectors), although the simple models considered in this paper implicitly assume spatial homogeneity by treating all parameters as population-level averages. Rabinovich and Himschoot [86], modeling host-vector contacts indirectly (through their effects on vector demographics) considered a gradual saturation in vector feeding due to irritability of both human and animal hosts (rather than the sharp saturation suggested here) with somewhat higher thresholds 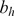 of 50 bites/host/night for reduced vector fecundity, 100 for starvation-induced mortality in nymphs, and 200 for starvation-induced mortality in adults, but these values are an order of magnitude higher than the observed ranges cited above.

We now return to the end goal of estimating 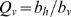 in light of our rough estimates for 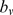 and the actual vector-to-host ratio 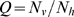. If 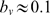 bites/vec/day and 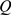 is about 14 for woodrats, 1600 for raccoons, and 3200 for opossums, then the question of whether in each case 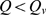 or 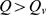 can be answered by estimating that probably 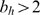 bites/host/night for woodrats, in which case 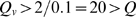, making vector-woodrat contacts saturated in hosts and therefore dependent on vector density, while certainly even as a very loose upper bound 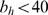 bites/host/night for raccoons and opossums, in which case 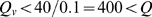, making vector-raccoon and vector-opossum bloodmeal contacts saturated in vectors even if the vector population is split between the two hosts, and therefore dependent on host population densities. (The sylvatic vector population may be split among more than just these two species of hosts, but the upper bound of 40 bites/host/night can also probably be reduced further.) This kind of indirect rough estimation is clearly less than ideal, but it is difficult to do better without direct data. At present the chief limiting factor in these estimations is the gross uncertainty in effective vector population density, with the density used in these calculations coming from a single source ([54], see derivation in Text S1). If the presence of additional host species besides the three mentioned here reduces the effective vector density (for contacts with these primary hosts) by an order of magnitude or more, some of the qualitative conclusions above regarding saturation may change.

## Discussion

Mathematical models have enormous predictive and explicative power in the study of biological systems, especially those where the feasibility of large-scale field studies is limited. Dynamical systems have managed to capture the nonlinear contact processes at the heart of many population biology questions in ecology and epidemiology, but their descriptive ability as models hinges on having accurate estimates for the biological parameters that measure key rates and quantities. Any study of the dynamics of sylvatic *Trypanosoma cruzi* infection must include both demographic and epidemiological information on the hosts and vectors involved.

As seen in the preceding sections and Text S1, a thorough literature review is sufficient to determine many of the most basic demographic parameters for the host and vector species that drive *T. cruzi* transmission in the southeastern quarter of the U.S., but many aspects of the contact processes which actually cause infection remain poorly understood. Simple dynamical systems models can be used to back-calculate infection rates from data on zoonotic prevalence, as well as to pinpoint what specific biological data needs to be gathered to complete parametrization of the models. In the present study, these data include: vector population densities, the probability of vertical transmission in raccoons and other hosts, the probability of oral infection per host type (and per vector consumed), the (maximum) rate at which hosts consume vectors, the extent to which *T. cruzi* infection changes the relevant behaviors of the vectors *T. sanguisuga* and *T. gerstaeckeri*, infection prevalence among Texas vectors outside woodrat nests and peridomestic sites, and the threshold vector-host density ratios which determine saturation for both contact processes.

The rough estimates derived in this paper regarding the latter ratios 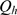 and 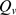 suggest that host predation on vectors is saturated in vectors (largely because this predation is opportunistic), and therefore dependent on host density for each host, whereas the vector feeding process is saturated in vectors only for the larger hosts (raccoons and opossums), which have a relatively low population density, and saturated in hosts for the woodrats that are the predominant host from central Texas south to Mexico, since woodrats occur at a higher density and return to the same nests on a long-term basis, making these nests efficient feeding sites for the vectors. Since *T. sanguisuga* and *T. gerstaeckeri* are widely believed to be inefficient vectors, the vector feeding process is primarily responsible for prevalence in vectors, and it is therefore interesting to note that *T. sanguisuga* appears to have a higher prevalence in many parts of the southeast (especially those closest to the center of the U.S.) than *T. gerstaeckeri* does in Texas, where it has ready access to abundant hosts. It is important to keep in mind, however, that the uncertainty in several parameter estimates (notably the effective vector population density) limits the confidence one can place in the conclusions regarding contact process saturation.

Of course, all models, however complex, remain caricatures or sketches of reality, and have their limitations. Dynamical systems models are limited in their predictive power not only by the accuracy of the estimates used for the biological rates that parametrize them, but also by the correctness and completeness of the assumptions that underlie every term in each equation. This paper is meant to connect these theoretical models to the many empirical studies that add detail to our understanding of the *T. cruzi* infection process in the U.S. Further studies are already in progress developing models that begin to incorporate the multiple infection mechanisms described in this work and the literature reviewed within, as well as the effects of dispersal and migration connecting the various evolving habitats (such as central Texas and the southeastern U.S.) where *T. cruzi* is in zoonosis. Readers interested in the question of vector feeding preferences for different types of host are referred to the studies [25] and [27].

## Supporting Information

Text S1 Demographic parameter estimates.
(0.09 MB PDF)
