# Supplementary material for: Estimating Contact Process Saturation in Sylvatic Transmission of Trypanosoma cruzi in the United States
Source: PLoS Negl Trop Dis. 2010 Apr 27;4(4):e656. doi: 10.1371/journal.pntd.0000656 (PMC2860507; doi:10.1371/journal.pntd.0000656)
Supplement: Text S1 — Demographic parameter estimates. (0.09 MB PDF) [file pntd.0000656.s001.pdf]

Estimating Contact Process Saturation in Sylvatic Transmission of *Trypanosoma cruzi* in the U.S. Supplementary Document

Christopher Kribs-Zaleta[1,*]
1 Christopher Kribs-Zaleta, Mathematics Department, University of Texas at Arlington, Arlington, TX 76019 USA
∗ E-mail: kribs@uta.edu

Demographic parameter estimates

Extensive research exists on many aspects of the demographics of sylvatic hosts and vectors of *T. cruzi* in the United States, but variations in geography, climate and the impact of humans and other predators cause a wide range of values to be reported for some of the parameters relevant to *T. cruzi* infection dynamics.

Host mortality and reproduction

Mortality rates (which we will denote by μ) can be estimated as the reciprocals of average lifespans. In Tennessee, Rabinowitz [1] recorded an average longevity for raccoons of 2.5 years and a maximum longevity of about 7.2 years. Annual mortality for adult raccoons has been reported at 40% in Iowa [2], which also corresponds ($\mu = 0.40/yr$) to an average lifespan ($1/\mu$) of 2.5 years, so we will use this value to estimate raccoon mortality rates. (Zeveloff reports variation in life expectancy of between 1.8 and 3.1 years, depending on factors such as hunting and climate [3].) Raccoons can live up to about 20 years in captivity, but food shortages, hunting by man, disease, and highway fatalities reduce the mean life expectancy to below 5 years in the wild (e.g., [4] as cited by [5]). Indeed, it is widely reported that 1/2 to 3/4 of the fall raccoon population consists of animals less than a year old.

Groce [5] cited an average life expectancy for opossums in the wild as less than 1.3 to 7 years. Olsen et al. [6] reported that opossums can live 1.33 years, although most probably live considerably less. Krause and Krause [7] reported that of those young opossums that survive weaning and go out on their own, fewer than one tenth live more than a year, and almost none live more than three years. This gives an upper bound of $(90\% \times 1) + (10\% \times 3) = 1.2$ years for life expectancy. We will take this value since it is close to the other figures above; this yields a mortality rate of $\mu = 0.83/\text{yr}$.

Adult male woodrats were observed in one mark-recapture study to live an average of 5.6 months after initial capture (as adults), while females survived an average 7.6 months ([8] and references therein). However, they may live 2 years or more, especially in captivity. Taking a time to maturity of about 0.4 years, this gives an overall lifespan of approximately one year ($\mu = 1/\text{yr}$).

Raccoons breed annually, with litter sizes reported as varying between 2 and 7 young. One study in Nebraska [2] found an average size of 4 young per litter born to females more than a year old, but an average of 3.3 young per litter born to yearling females (female raccoons become sexually mature at one year of age). Reporting also that 95% of older females bred, while only 70% of yearling females bred, this yields an average of 3 young per sexually mature year of life. Another study in Cades Cove, Tennessee, an isolated valley in the Great Smoky Mountains, found an average litter size of 2.8 young [1]. (More broadly, Zeveloff [3] reports that average litter sizes vary from 2.5 in Alabama to 4.8 in North Dakota.) To estimate the maximal reproductive rate g for raccoons, therefore, we multiply

$$g = 3\,\frac{\text{raccoons}}{\text{litter}} \times 1\,\frac{\text{litter}}{\text{raccoon}\cdot\text{s.m.yr}} \times \frac{1.5\text{ s.m.yr}}{2.5\text{ yr}} \times \frac{1}{2} = 0.9/yr,$$

where s.m.yr stands for sexually mature year(s), the third factor above represents the proportion of a

female's lifetime during which she is reproductively mature, and the final factor of 1/2 takes into account an assumed 1:1 sex ratio, included here since only females bear young.

Opossums mate twice per year; although as many as 16 young may be born at a time, mothers are generally only able to nurse and raise about 8 (see references in [5]). Female opossums become sexually mature at around six months of age. Using the same approach as for raccoons above, this yields an estimated maximal growth rate of

$$g = 8\,\frac{\text{opossums}}{\text{litter}} \times 2\,\frac{\text{litters}}{\text{opossum} \cdot \text{s.m.yr}} \times \frac{0.7 \text{ s.m.yr}}{1.2 \text{ yr}} \times \frac{1}{2} = 4.7/yr.$$

This estimate agrees precisely with the postnatal growth rate given for *D. virginiana* (fit to a Gompertz function) of 0.0129/day given in [9].

Female woodrats reach sexual maturity at around 136 days. Their average litter size is 2.51 and they have a mean of 2.4 litters per year [9]. If we suppose that a female woodrat is sexually mature for about 60% of her lifetime (mean time to sexual maturity 136 days, mean lifespan 1 year), the maximal woodrat growth rate can be estimated by $g = 2.51 \times 2.4 \times 0.6 \times 0.5 = 1.8/yr$.

Host population density

Estimates for host population densities vary extremely widely (by orders of magnitude in some cases) by geography as well as by study, due to factors such as landscape, climate, urbanization, food abundance, and predators.

Sonenshine and Winslow [10] estimated one raccoon per 14.3 acre (.07 animals/acre) in a sylvatic setting in Virginia. Another source [11] estimates one raccoon per 30–40 acres (.025–.033 animals/acre) in river bottoms and agricultural areas near woodlands and waterways in Wisconsin, and one raccoon per 12–20 acres (.05–.083 animals/acre) in older residential areas with plenty of ground cover (and available food). Conner et al [12] reported a density of 10.3-11.4/km^2 (0.0417–0.0462 animals/acre, 1 km^2 ≈247 acres) for raccoons at a Welaka, Florida site composed primarily of pine flatwoods with some bottomland hardwoods adjacent to the St. Johns River, during winter 1981. Another study measuring winter densities at two hardwood-diminated sites in the Tennessee hills estimated one raccoon per 70.4 ha (.00575 animals/acre) at one site and one raccoon per 34.5 ha (.0117 animals/acre) at the other [13]. Zeveloff [3] reports densities of 0.5 to 3.2 animals/km^2 (.002 to .013 animals/acre) in prairies, up to 6 animals/km^2 (.024 animals/acre) in upland hardwood forests, and over 20 animals/km^2 (.08 animals/acre) in lowland forests and marshes. Urban [14] reported a density of 45.3 raccoons/sq. mile (0.0708 animals/acre) in a marsh on Lake Erie. Broadfoot et al. [15] estimated a present (1987–1996) density of 15 raccoons/km^2 (0.061 animals/acre) and an equilibrium (eventual) raccoon density of 23.5 animals/km^2 (0.0951 animals/acre) in the urban environment of Scarborough, Ontario; excluding area where raccoons cannot reside (such as office buildings), the latter density estimate rises even further, to 53 raccoons/km^2 (they projected carrying capacities of 54–62 animals/km^2, with a median of 57).

There are, however, several other significantly higher estimates: Riley et al.'s [16] estimate cited in [5] that in human-dominated areas raccoon population density may range from 40 to 120 animals/km^2 (0.16–0.49 animals/acre) seems quite high by comparison, as does their overall national park estimate of 125 animals/km^2 (0.506 animals/acre). Hoffmann and Gottschang [17] reported a density of one raccoon per 1.46 ha (0.278 animals/acre) in a 1973–1974 study of Glendale, Ohio, a Cincinnati suburb. The variation may be explainable by variation in time of year or environment: Blackwell et al. [18] calculated an average raccoon density of 24.5 animals/km^2 (0.0992 animals/acre) from August to November on a NASA property in Erie Co., Ohio consisting of fragmented forest and old-field landscapes as well as some industrial buildings, although their study found a marked decrease from August (33.4 raccoons/km^2, 0.135 animals/acre) through November (13.6 raccoons/km^2, 0.0551 animals/acre). Prange et al. [19] reported average densities of roughly 40–70 raccoons/km^2 (0.16–0.28 animals/acre) in an urban setting, 40–90 raccoons/km^2 (0.16–0.36 animals/acre) in a suburban setting, and roughly 4–16 raccoons/km^2

(0.016–0.065 animals/acre) in a rural setting. Since we are interested in primarily sylvatic sites, with wooded landscapes in the American southeast, we will estimate an average density of 20. animals/km^2 (.08 animals/acre), consistent with the range of densities reported for many of the rural habitats studied.

References in [5] and [6] reported opossum population densities of 1 animal per 1/2 acre, per 2.75 acres in Mississippi and Illinois, per 4 acres in east Texas, per 14.6 acres along the coast, per 20 acres in Kansas farmland, per 30 acres in upstate New York, and per 64 acres on cultivated land in northwestern Illinois. Stout and Sonenshine [20] estimated an average of one opossum per 50 acres on a site in Virginia during 1963–1969. Conner et al [12] reported a density of 10.1/km^2 (0.0409 animals/acre, or one opossum per 24.5 acres) for opossums at the Welaka, Florida site mentioned above during winter 1981. Kissell and Kennedy [13] estimated one opossum per 9.9 ha (0.0409 animals/acre) at one wooded site in the Tennessee hills and one opossum per 44.5 ha (0.00909 animals/acre) at another, both during winter as well. Despite winter population densities being the lowest of the year, the two equivalent estimates of 0.0409 opossums/acre is representative of all but the three highest estimates given here (which are 6–50 times as high), so we shall take this as a working average density.

Estimates for woodrat (*N. micropus*) density include 18.5 woodrats/hectare (ha) and 29.7 woodrats/ha in desert-scrub habitat, and another study in which the density in a single area during a 2.5-year period was as high as 31.1 woodrats/ha and ended at zero when 90% of the cacti died due to excessive rainfall, with the longest single period of equilibrium (10 months) corresponding to a density of 24.7 woodrats/ha (see [8] and references therein). Russell et al. [21] estimated a density of 19.4 woodrats/ha on a brushy site outside San Antonio, Texas, although an earlier study outside Alice, Texas (about 130 miles south of the other site) had reported no more than 1.5 woodrats/ha. A study of dusky-footed woodrats (*Neotoma fuscipes*) in California found an average density of 81.12 animals/ha in sapling/brushy poletimber stands (but less than 1 per acre among seedlings and old growth forest) [22]; it is not clear how much of this difference can be attributed to the difference in species, and how much to the different landscapes. For the scrub-dominated landscapes of central and south Texas, we shall assume an average density of 23 woodrats/ha (9.3 animals/acre), which is the mean value of the four *N. micropus* densities reported above.

A population governed by logistic growth and linear per-capita mortality,

$$\frac{dN}{dt} = gN\left(1 - \frac{N}{K}\right) - \mu N,$$

approaches an equilibrium density N^* below the habitat's carrying capacity K, in particular $N^* = K(1 - \mu/g)$ (assuming $\mu < g$). (This model is mathematically equivalent to a simple logistic model $\frac{dN}{dt} = rN\left(1 - \frac{N}{N^*}\right)$ with $r = g - \mu$.) Therefore, if we take the density estimates above to represent equilibrium densities, we can back-calculate carrying capacities as $K = N^*/(1 - \mu/g)$. Using the estimates given above results in carrying capacities of 0.144 raccoons/acre, 0.0497 opossums/acre, and 21 woodrats/acre.

Vector mortality and reproduction

Hays [23] reported a mean longevity for *T. sanguisuga* of 456.5 days for females and 526 days for males, under laboratory conditions. (These figures are for adult insects born in the wild and collected as nymphs: Hays found that laboratory-born *T. sanguisuga* lived only 215 and 228 days, respectively.) Pippin [24] reported average longevities of 527.4 days for male adults and 589.2 days for female adults of *T. s. texana* under variable temperatures and humidities (18–28°C, 20–65% relative humidity) in the laboratory; for constant conditions (27°C, 65% relative humidity) the means were 625.6 days and 516.4 days for male and female adults, respectively. The mean developmental period (from egg to adult) was 387.8 days with fluctuating temperatures and 322.1 days at the constant temperature. However, under field conditions it took *T. s. texana* nymphs over two years to mature (the minimum reported times were 715 days (2 years) for one nymph and 2.5 years for two others), and Pippin suggested a 24 to 30 month life cycle for *T. s. texana* in southwest Texas. Taking an average field maturation time of 2.25 years and an average

adult lifespan of 525 days (the mean of the first two data given above from Hays and the first two data given above from Pippin) or 1.44 years gives a total average lifespan ($1/\mu$) of 3.69 years.

Thurman [25] reported that *T. gerstaeckeri* adults lived 6–11 months in the laboratory. Consistent with this range, Pippin [24] reported average longevities of 285.4 days for male adults and 285.0 days for female adults of *T. gerstaeckeri* under variable temperatures and humidities (18–28°C, 20–65% relative humidity) in the laboratory; for constant conditions (27°C, 65% relative humidity) the means were 317.4 days and 312.6 days for male and female adults, respectively. The mean developmental period was 361.9 days with fluctuating temperatures and 213 days under constant temperature. The minimum developmental period under field conditions, however, was 280 days, and Pippin suggested a life cycle for *T. gerstaeckeri* in southwest Texas of 9 to 14 months. Martínez-Ibarra et al. [26] found a mean developmental time (egg to adult) of 278.6 days under constant laboratory conditions. We shall use Pippin's mean developmental period of approximately 1 year, and the mean adult lifespan under variable laboratory conditions of 285.2 days (0.78 years), to estimate a total ($1/\mu$) of 1.78 years.

Olsen et al. [6] and Hays [23] reported an average of 711 eggs laid per female *T. sanguisuga* under laboratory conditions, and an average hatching percentage of 77.3%. Pippin [24] found an average of 712.6 eggs per *T. s. texana* female and an average hatching proportion of 65.8%, also in the laboratory. These figures were significantly lower in the field: a mean ($n = 3$) of 465 eggs laid in a year, of which only 279 (60.0%) hatched. However, if we multiply 465 eggs/yr by the expected adult lifetime of 1.44 years we get 670 eggs, which is not far off the 711–712 eggs cited above. Since none of these studies reported rates of survivorship to adulthood (post hatching) we shall take the figure of 45/75=60% reported (see next paragraph) for *T. gerstaeckeri.* Now we can calculate the maximal growth rate for *T. sanguisuga* as follows:

$$g = 465\,\frac{\text{eggs}}{\text{s.m.yr}\cdot\text{female}} \times \frac{1.44\,\text{s.m.yrs}}{3.69\,\text{yr}} \times 0.60\,\frac{\text{hatch}}{\text{egg}} \times 0.60\,\frac{\text{adult}}{\text{hatch}} \times \frac{1\,\text{female}}{2\,\text{adult}} = 33/yr.$$

Pippin [24] found an average of 675.4 eggs per *T. gerstaeckeri* female and an average hatching proportion of 71.2%, also in the laboratory. In the field the figure was 803 eggs, of which 485 (60.3%) hatched. Martínez-Ibarra et al. [26] found an average hatching proportion of 75.0% in the laboratory (but only 45% of the eggs hatched nymphs that survived to adulthood) and cited an earlier study which had reported a 66.9% hatch rate. These data support a mean 71% hatch rate, although we shall use the net 45% survivalship to adulthood which incorporates hatching proportion (the same authors found a similar survival rate—40%—for *Triatoma pallidipennis*). Thus we calculate, using the mean lifetime estimated above,

$$g = 803\,\frac{\text{eggs}}{\text{female}} \times \frac{1}{1.78\,\text{yr}} \times 0.45\,\frac{\text{adult}}{\text{egg}} \times \frac{1\,\text{female}}{2\,\text{adult}} \approx 100/yr.$$

Vector population density

A literature review revealed only one estimate for sylvatic triatomine densities in North America (and density estimates in South America focused exclusively on domestic, per building counts). Burkholder et al. [27] estimated a summer density of 247 *Triatoma* sp. per hectare in wooded areas of Cameron Co. and 384 per hectare in similar areas of Hidalgo Co., Texas. We will use the mean of these two figures, 315.5 vectors/ha (128 vectors/acre), as the equilibrium densities for both vector species, despite variations in landscape and climate which are likely significant. In order to achieve this density, given the reproduction and mortality figures above, one needs carrying capacities of 129 vectors/acre (the difference for the two species is less than the precision of the data since for both species $\mu/g < 0.01$).

Another study with relevant data, although it does not directly provide for a population density estimate, is that of Pippin [24], who captured 698 adult *T. sanguisuga* and *T. gerstaeckeri* vectors over two summers (1966 and 1967) near San Antonio, Texas using blacklight traps at two sites at night. Given the long life cycle of the vectors (e.g., mean developmental times of about a year for both species

as discussed above), the adult vectors captured the second summer must already have been alive during the first summer, so that Pippin captured approximately 350 vectors per site from an existing population. Although it is not possible to know how far each vector traveled to the traps, some studies of flight in South American triatomine species have estimated representative flight ranges of around 200m [28]. If we suppose (ignoring the many other differences in vector species and climate between the two settings) that the vectors captured in the traps originated within a 200m × 200m area, Burkholder et al.'s density estimate would suggest that the 350 vectors came from a population in that area of about 1264 vectors, implying that the blacklight traps caught more than 1/4 of the area's population. At the very least, this suggests that Burkholder et al.'s estimate is not off by orders of magnitude.

Since our interest in estimating population densities is to determine the frequency of host-vector contacts, it is also worth observing that triatomines tend to remain highly localized in hosts' sleeping places, rather than spread uniformly over the landscape. Since each host has multiple sleeping places and only visits one per night, on any given night that host encounters only a fraction of the vectors within its territory. However, by the same token the vectors in that sleeping place, which have been without a host on which to feed for several nights while the host was sleeping elsewhere, will then feed at a higher rate than if the host returned to that sleeping place every night, with the result that the host does effectively contact all of the vectors in its territory, at a rate high enough to sustain the bug population there. Therefore we will not alter the estimated population densities in response to this spatial heterogeneity of the vectors (and corresponding itinerant sleeping habits of the hosts).

Table 1 in the main text, reproduced below, summarizes these estimates (including SI equivalents) for the demographic parameters of each species.

Table 1. Estimates for demographic parameters

**Species**	**Death rate** μ	**Growth rate** g	**Density carrying capacity** K	**Equilibrium density** $N^* = K\left(1 - \frac{\mu}{g}\right)$
Raccoon	0.40/yr	0.9/yr	0.144 rac/acre (35.6 rac/km^2)	0.08 rac/acre (20. rac/km^2)
Opossum	0.83/yr	4.7/yr	0.0497 opo/acre (12.3 opo/km^2)	0.0409 opo/acre (10.1 opo/km^2)
Woodrat	1/yr	1.8/yr	21 rats/acre (5200 rats/km^2)	9.3 rats/acre (2300 rats/km^2)
T. sanguisuga	0.271/yr	33/yr	129 vec/acre (31900 vec/km^2)	128 vec/acre (31600 vec/km^2)
T. gerstaeckeri	0.562/yr	100/yr	129 vec/acre (31900 vec/km^2)	128 vec/acre (31600 vec/km^2)

References

1. Rabinowitz AR (1981) The ecology of the raccoon (*Procyon lotor*) in Cades Cove, Great Smoky Mountains National Park. Ph.D. thesis, University of Tennessee, Knoxville, TN.

2. Nebraska Game and Parks Commission (n.d.). Nebraska wildlife species guide. URL `http://www.ngpc.state.ne.us/wildlife/raccoon.asp`. Accessed 20 April 2009.

3. Zeveloff SI (2002) Raccoons: a natural history. Washington, D.C.: Smithsonian Books.

4. Lotze JH, Anderson S (1979) *Procyon lotor*. Mammalian Species 119: 1–8.

5. Groce BC (2008) *Trypanosoma cruzi* in wild raccoons and opossums from Kentucky. Master's thesis, Western Kentucky University.

6. Olsen PF, Shoemaker JP, Turner HF, Hays KL (1966) The epizoology of Chagas' disease in the southeastern United States. Wildlife Disease 47: Suppl. 1–108.

7. Krause WJ, Krause WA (n.d.) The opossum: its amazing story. University of Missouri School of Medicine. URL `http://web.missouri.edu/~krausew/Histology/ Home_files/opossum.pdf`. Accessed 20 April 2009.

8. Braun JK, Mares MA (1989) Neotoma micropus. Mammalian Species 330: 1–9.

9. Human Ageing Genomic Resources (Steven Austad, curator). The animal ageing and longevity database. `http://genomics.senescence.info/species/index.html`. Accessed 20 April 2009.

10. Sonenshine DE, Winslow EL (1972) Contrasts in distribution of raccoons in two Virginia localities. Journal of Wildlife Management 36: 838–847.

11. Wisconsin Department of Natural Resources (n.d.). Raccoons. URL `http://www.dnr.state.wi.us/org/land/wildlife/PUBL/wlnotebook/FSRaccoon.htm`. Accessed 20 April 2009.

12. Conner MC, Labisky RF, Progulske Jr DR (1983) Scent-station indices as measures of population abundance for bobcats, raccoons, gray foxes, and opossums. Wildlife Society Bulletin 11: 146–152.

13. Kissell Jr RE, Kennedy ML (1992) Ecologic relationships of co-occurring populations of opossums (*Didelphis virginiana*) and raccoons (*Procyon lotor*) in Tennessee. Journal of Mammalogy 73: 808–813.

14. Urban D (1970) Raccoon populations, movement patterns, and predation on a managed waterfowl marsh. Journal of Wildlife Management 34: 372–382.

15. Broadfoot JD, Rosatte RC, O'Leary DT (2001) Raccoon and skunk population models for urban disease control planning in Ontario, Canada. Ecological Applications 11: 295–303.

16. Riley SPD, Hadidian J, Manski DA (1998) Population density, survival, and rabies in raccoons in an urban national park. Canadian Journal of Zoology 76: 1153–1164.

17. Hoffmann CO, Gottschang JL (1977) Numbers, distribution, and movements of a raccoon population in a suburban residential community. Journal of Mammalogy 58: 623–636.

18. Blackwell BF, Seamans TW, White RJ, Patton ZJ, Bush RM, et al. (2004) Exposure time of oral rabies vaccine baits relative to baiting density and raccoon population density. Journal of Wildlife Diseases 40: 222–229.

19. Prange S, Gehrt SD, Wiggers EP (2003) Demographic factors contributing to high raccoon densities in urban landscapes. Journal of Wildlife Management 67: 324–333.

20. Stout IJ, Sonenshine DE (1974) Ecology of an opossum population in Virginia, 1963–69. Acta Theriologica 19: 235–245.

21. Raymond RW, McHugh CP, Witt LR, Kerr SF (2003) Temporal and spatial distribution of *Leishmania mexicana* infections in a population of *Neotoma micropus*. Memorias do Instituto Oswaldo Cruz 98: 171–180.

22. Sakai HF, Noon BR (1993) Dusky-footed woodrat abundance in different-aged forests in northwestern California. Journal of Wildlife Management 57: 373–382.

23. Hays KL (1965) Longevity, fecundity, and food intake of adult *Triatoma sanguisuga* (Leconte) (Hemiptera: Triatominae). Journal of Medical Entomology 2: 200–202.

24. Pippin WF (1970) The biology and vector capability of *Triatoma sanguisuga texana* Usinger and *Triatoma gerstaeckeri* (Stål) compared with *Rhodnius prolixus* (Stål) (Hemiptera: Triatominae). Journal of Medical Entomology 7: 30–45.

25. Thurman Jr DC (1945) Biology of *Triatoma gerstaeckeri*. Journal of Economic Entomology 38: 597–598.

26. Martínez-Ibarra JA, Alejandre-Aguilar R, Paredes-González E, Martínez-Silva MA, Solorio-Cibrián M, et al. (2007) Biology of three species of North American Triatominae (Hemiptera: Reduviidae: Triatominae) fed on rabbits. Memorias do Instituto Oswaldo Cruz 102: 925–930.

27. Burkholder JE, Allison TC, Kelly VP (1980) *Trypanosoma cruzi* (Chagas) (Protozoa: Kinetoplastida) in invertebrate, reservoir, and human hosts of the Lower Rio Grande Valley of Texas. Journal of Parasitology 66: 305–311.

28. Schofield CJ, Lehane MJ, McEwen P, Catalá SS, Gorla DE (1992) Dispersive flight by *Triatoma infestans* under natural climatic conditions in argentina. Medical and Veterinary Entomology 6: 51–56.
